# Association of serum levels of Visfatin, Intelectin-1, RARRES2 and their genetic variants with bone mineral density in postmenopausal females

**DOI:** 10.3389/fendo.2022.1024860

**Published:** 2022-11-30

**Authors:** Sundus Tariq, Saba Tariq, Saba Khaliq, Shahad Abduljalil Abualhamael, Mukhtiar Baig

**Affiliations:** ^1^ Department of Physiology, University Medical & Dental College, The University of Faisalabad, Faisalabad, Pakistan; ^2^ Department of Pharmacology and Therapeutics, University Medical & Dental College, The University of Faisalabad, Faisalabad, Pakistan; ^3^ Department of Physiology and Cell Biology, University of Health Sciences, Lahore, Pakistan; ^4^ Department of Internal Medicine, Faculty of Medicine Rabigh, King Abdulaziz University, Jeddah, Saudi Arabia; ^5^ Department of Clinical Biochemistry, Faculty of Medicine, Rabigh, King Abdulaziz University, Jeddah, Saudi Arabia

**Keywords:** BMD, osteoporosis, intelectin-1, RARRES2, adipokines, visfatin/NAMPT

## Abstract

**Background:**

Adipokines are engaged in bone physiology and regulate bone mineral density (BMD) by playing protective or cynical role in bone metabolism. The study is designed to measure and compare BMD, adipokines (retinoic acid receptor responder protein-2 RARRES2, visfatin and Intelectin-1) and their genetic variants in postmenopausal osteoporotic, osteopenic and non-osteoporotic females.

**Methods:**

This comparative study included postmenopausal non-osteoporotic (n=72), osteopenic (n=72) and osteoporotic (n=100) females with two years of amenorrhea and age between 50 to 70 years. Gold standard DXA was used to measure BMD. Hardy-Weinberg equilibrium was established. Kruskal-Wallis test for comparisons, logistic and multivariate regression analysis were used to rule out the predictors of BMD.

**Results:**

On comparing the three groups, significant differences were observed in serum RARRES2 (*p <*0.001) and serum visfatin (*p*=0.050). The significant positive predictor of BMD at lumbar spine and total hip was serum visfatin. BMD at right and left femoral neck was predicted negatively by serum chemerin while BMD at left femoral neck was also predicted positively by serum calcium levels. There was significant difference in BMD at right femoral neck (*p* = 0.033) between rs7806429 genotypes. The odds of having low BMD increases with increasing serum levels of chemerin and decreasing serum levels of visfatin and calcium

**Conclusion:**

The adipokines RARRES2 and visfatin are associated with BMD. RARRES2 is an independent negative and visfatin is positive predictor of BMD in postmenopausal females. BMD at right femoral neck was significantly low in RARRES2 rs7806429 TC heterozygotes.

## Introduction

Osteoporosis is a multifactorial disease. It is initiated by complex interplay between various environmental, metabolic and genetic factors, leading to microarchitecture and mechanical deterioration of bones and resultant fragility fractures. Recently, research has shown the relation of adipokines with the bone mineral density (BMD) in the osteoporotic subjects (1). The adipokines (adipocytokines) are chemical messengers, which were previously thought to be released by the adipocytes only, are expressed by many tissues of the body and play a versatile part in various physiological aspects of the body including bone metabolism (2).

Retinoic acid receptor responder protein 2 (RARRES2) also called chemerin is a 14 kDa protein. It acts as a ligand for chemokine like receptor-1 (CMKLR1), a G protein coupled serpentine receptor. *RARRES2* gene has six exons and is located on chromosome 7q36.1 (3). RARRES2 has a role in inflammation and adipocyte differentiation (4). It has a negative correlation with BMD and its serum levels are significantly raised in osteoporotic patients (5). It seems that it has a facilitating role in adipogenesis but inhibitory role in osteogenesis (6). In contrast, another study showed significantly decreased levels in postmenopausal osteoporotic females. However, no plausible cause of this finding was documented (7).

Single nucleotide variations in *RARRES2* gene were studied in relation to various diseases but not osteoporosis. Genome wide meta-analysis showed strong association of *RARRES2* rs7806429 single nucleotide variant (SNV) with circulating RARRES2 concentrations (8). We selected *RARRES2* rs7806429 SNV, to observe its association with BMD and serum RARRES2 levels, which may further add in our understanding of the role of RARRES2 as potential biomarker for osteoporosis in postmenopausal females.

Visfatin also known as pre-B-cell colony-enhancing factor 1 (PBEF1) with full name of nicotinamide phosphoribosyl transferase (NAMPT) belongs to nicotinic phosphoribosyl transferase family and plays an important role in metabolism, aging and stress response. Its gene is located on chromosome 7q22.3 with an exon count of 12 (9). Human serum levels of visfatin are involved in anabolic activity of bone metabolism (10). Visfatin is believed to exert its anabolic effects on bone by altering the differentiation and functional activity of osteoblasts (11). Contrasting findings elaborating the catabolic action of visfatin on bone metabolism are also documented (12, 13). The research done so far on the potential role of visfatin related to bone is inconclusive. This indistinct division among anabolic and catabolic impacts hamper any transient utilization of visfatin as a potential marker for the diagnosis and treatment of osteoporosis. Therefore, further research is required on this front to determine the use of visfatin related to osteoporosis and diseases related to bone metabolism.

Visfatin rs2302559 polymorphism was associated with serum visfatin levels (14). As literature has shown the relation of serum visfatin levels with BMD and no study has explored the role of visfatin gene polymorphisms in relation to BMD. So, association of SNV rs2302559 has also been evaluated with BMD in this study.

Intelectin-1 (ITLN-1) also called omentin-1 is derived from the omental adipose tissue and encoded by *ITLN-1* gene located on chromosome 1q23.3 and consists of eight exons and seven introns (15). It was found to have a negative role in osteoblastic differentiation and has a negative correlation with BMD (16). A study exploring the frequency of polymorphism 326A/T of gene *ITLN-1* has shown that women with osteoporosis and osteopenia homozygous for AA genotype may lead to lower BMD (17).

Internationally, there are scanty, controversial and opposing data available regarding the relation of these novel adipokines (RARRES2, Intelectin-1 and visfatin) with BMD, and absolutely no published data from local population. Furthermore, other than intelectin-1 gene polymorphism 326A/T in postmenopausal Polish population (17), no polymorphisms of the selected genes have been described from local or any other population of postmenopausal females, internationally.

The short review above clearly shows that the information regarding adipokines narrated is quite scanty and equivocal when available. Therefore, present study is designed to find out serum levels of these adipokines (RARRES2, intelectin-1, visfatin) in postmenopausal osteoporotic, osteopenic and healthy postmenopausal females. In addition, genetic polymorphisms, in the genes regulating their activity were also studied in groups mentioned above. The results of this study may help us to delineate the role of these adipokines in the overall bone metabolism in postmenopausal women.

## Materials and methods

The comparative study was conducted on 244 postmenopausal females after taking written informed consent. The study was conducted according to the guidelines of the Declaration of Helsinki, and approved by the Ethics Committee of University of Health Sciences, Lahore. The study subjects were recruited from Madina Teaching Hospital, following the inclusion and exclusion criteria. Postmenopausal females between the age of 50 to 70 years and two years of amenorrhea were included. Females with T-score -1.0 or higher, -1.0 to -2.5 and -2.5 or lower, measured through DXA according to WHO densitometric crtiteria (18), were placed in non-osteoporotic (n=72), osteopenic (n=72) and osteoporotic (n=100) groups respectively. Postmenopausal females with chronic renal disease, chronic liver disease, malignancies, autoimmune disease, parathyroid hormone problems, iatrogenic or premature menopause and taking medications like steroids, vitamin D or bisphosphonate therapy were not included in the study.

### Bone densitometry

Bone mineral density (BMD) of postmenopausal females was evaluated by HOLOGIC-HORIZON (QDR-series), dual energy X-ray absorptiometry (DXA) system at Pakistan Institute of Nuclear Medicine (PINUM) Hospital, Faisalabad. BMD was evaluated at the lumbar spine (L2-L4), right femoral neck, left femoral neck and total hip. The results of DXA were used for final analysis and presented as T-scores. According to the criteria set by world health organization (WHO), osteoporosis in adults is diagnosed by the T-scores obtained from DXA. T-score is defined as, the comparison of measured BMD result with the average BMD of the young adults at the time of peak bone mass. T-score ≤ 2.5 standard deviations below the mean peak bone mass represent osteoporosis.

### Biochemical analysis

Blood samples were collected from the subjects after an overnight fast. Four mL blood was dispensed in vacutainer without any anticoagulant, centrifuged (3000 rpm or 1000xg for 10 min) after two hours to separate the serum and stored at -70 °C until used. The biochemical and genetic analysis were performed at the postgraduate research laboratory of The University of Faisalabad.

Serum calcium, phosphate and alkaline phosphatase were measured by colorimetric method, using Roche diagnostics, cobas 6000, COBI-CD, manufactured by Hitachi high technologies corporation, Tokyo, Japan. Serum adipokines (RARRES2, Intelectin 1, Visfatin) were assessed quantitatively by ELISA using kits formulated by Elabscience Biotechnology Inc. Houston, Texas 77079, USA, and quantified by microplate data collection and analysis software Gen5™ and Gen5 Secure, manufactured by BioTek^®^ Instruments, Inc. Winooski, Vermont 05404-0998, USA. The coefficient of variation for all assays were <10%.

### Genotyping

DNA from whole blood was extracted using GeneJet whole blood genomic DNA purification mini kit, manufactured by Thermo Fisher Scientific Inc. Carlsbad, California 92008, USA. The integrity and quality of extracted DNA was assessed by using 1.0% agarose gel electrophoresis. Primers were designed, by using Tetra-primer ARMS-PCR tool and Primer3Plus tool. All the designed primers were then BLAST in the bioinformatics tool to determine homology and avoid mismatch and secondary structure formation within the primers.

*RARRES2*

rs7806429-F1TGGGCCTGTTTAAACTTTTTATTCCAGTT
rs7806429-F2GCAATGTATTGACAGCCAGATTAAGGG
rs7806429-R1TGAATGCCTAATATTCCATCATGTGGAT
rs7806429-R2TCAGATAAAGAATAGTAGTGGCTGGGGG

*PBEF1*

rs2302559-F1TATTAGTGGTGCCTGTGTACTTCGC
rs2302559-F2TATTAGTGGTGCCTGTGTACTTCGT
rs2302559-RGGTGATTCAGGGATAGATCA

*ITLN-1*

rs2274907-FGAGCCTTTAGGCCATGTCTCT
rs2274907-RCTCTCTTCTTCTCCAGCCCAT

The genetic regions of different genes were amplified from extracted DNA using gene specific primers under optimized conditions. After PCR amplifications, analysis of selected genetic variations were done by restriction fragment length polymorphism (RFLP)/amplification refractory mutation system (ARMS)/Allele specific PCR. Amplicons were checked using agarose gel electrophoresis. After PCR about 10 µl of amplicon was incubated with restriction enzymes. Digested samples were subjected to agarose gel electrophoresis to identify digestion patterns. The results of PCR for few single nucleotide variants (SNVs) were confirmed by sequencing. Samples were sequenced by Advance Bioscience International (ABI) China. The data obtained after sequencing were then visualized by using Chromas software to find out the variations in these sequences. Samples with ambiguous results were excluded from the final analysis. The sample size for genetic analysis was n = 243 for *RARRES2* rs7806429, n = 242 for *PBEF1* rs2302559 and n = 243 for *ITLN-1* rs2274907.

### Statistical analysis

Data were analyzed by SPSS version 26.0 (Statistical Package for Social Sciences). Normality of the data was checked by Shapiro-Wilk’s statistics. Groups were compared using Kruskal-Wallis test. *Post-hoc* pairwise comparisons were performed using Dunn-Bonferroni approach. Multivariate linear stepwise regression and logistic regression analysis was used to predict the BMD. The independent variables used in the model were serum RARRES2, intelectin-1, visfatin, calcium, phosphate and alkaline phosphatase. The model was adjusted for age and menopausal age. In multivariate analysis, a forward stepwise regression approach was adopted. The model added the most significant variables with the highest p-value first followed by the less significant variables. For logistic regression, BMD was included as dichotomous categorical variable. The diseased group has low BMD (including both osteopenic and osteoporotic subjects) while the non-diseased group was represented with subjects having normal BMD. The samples with any missing data were not included in the analysis. Genotypic, allelic frequencies and Hardy-Weinberg equilibrium were calculated. Association of various biochemical markers and BMD with genotype frequencies were observed using Kruskal-Wallis test. P-value ≤ 0.05 was taken as statistically significant.

## Results

The study included 244 postmenopausal females, divided into three groups, Normal (n=72), Osteopenic (n=72) and Osteoporotic (n=100). The study groups were age matched. The comparison of general characteristics and biochemical parameters are presented in **Table 1**. Significant differences were observed between the serum levels of RARRES2 (*p <*0.001) and visfatin (*p* = 0.050), while no significant difference was observed in the serum levels of other parameters (**Table 1**).

**Table 1:** Comparison of general characteristics and biochemical parameters of the study population.

Variables	Normal (n=72)	Osteopenic (n=72)	Osteoporotic (n=100)	*p*-value*
Median (IQR)				
**Age (years)**	56.50 (50.25-64.00)	57.00 (54.00-63.00)	57.00 (53.25-65.00)	0.090
**Menopausal age (years)**	50.50 (49.00-54.00)	50.00 (46.00-53.00)	50.00 (48.00-52.75)	0.060
**Chemerin (ng/mL)**	0.14 (0.08-0.26)	0.15 (0.13-0.22)	0.24 (0.13-0.52)	<0.001*^a^
**Visfatin (ng/mL)**	7.57 (2.86-12.95)	6.21 (2.84-13.07)	4.96 (1.82-9.41)	0.050*^b^
**Intelectin-1 (ng/mL)**	6.54 (1.75-14.02)	9.28 (3.18-18.11)	4.43 (1.73-12.38)	0.144
**Alkaline Phosphatase (U/L)**	93.00 (77.50-112.75)	98.00 (78.25-116.00)	99.00 (80.00-124.00)	0.316
**Calcium (mg/dL)**	9.60 (9.40-9.80)	9.50 (9.30-9.70)	9.50 (9.20-9.90)	0.152
**Phosphate (mg/dL)**	3.80 (3.40-4.10)	3.80 (3.40-4.10)	3.80 (3.40-4.20)	0.948

*p-value ≤ 0.050 is considered statistically significant. ^a^ Significant differences were observed between normal – osteoporotic and osteopenic –osteoporotic groups. ^b^ Significant differences were observed between normal-osteopenic and normal-osteoporotic groups.

Multivariate linear stepwise regression analysis was used to predict the BMD at all sites. The independent variables used in the model were serum RARRES2, intelectin-1, visfatin, calcium, phosphate and alkaline phosphatase. The model was adjusting for age and menopausal age. At the lumbar spine and hip, the independent positive predictor was serum visfatin. At right femoral neck, the independent negative predictor was serum chemerin while at left femoral neck, the independent negative predictor was serum chemerin and positive predictor was serum calcium (**Table 2**).

**Table 2:** Stepwise multiple regression analysis showing independent predictors of BMD.

Dependent	Model	Predictors	β	SE- β	95% CI for β	*p-*value
Lumbar spine	1	Visfatin	0.008	0.003	0.002 – 0.013	0.005*
Right femoral neck	1	Chemerin	-0.300	0.120	-0.537 – -0.063	0.013*
Left femoral neck	1	Chemerin	-0.301	0.116	-0.530 – -0.071	0.010*
	2	Calcium	0.319	0.154	0.015 – 0.623	0.040*
Total hip	1	Visfatin	0.005	0.002	0.001 – 0.010	0.018*

Dependent variable is BMD (T-scores) Independent variables (predictors/covariates) used in the model are serum chemerin, intelectin-1, visfatin, calcium, phosphate and alkaline phosphatase Model is adjusted for age and menopausal age * p-value ≤ 0.050 is considered statistically significant.

A logistic regression analysis was performed to ascertain the effects of serum visfatin, Intelectin-1, chemerin, alkaline phosphatase, calcium and phosphate on the likelihood that participants have low BMD (osteopenia and osteoporosis). Model was adjusted for age and menopausal age. The logistic regression model was statistically significant, χ2 (8) = 34.202, p < 0.001. The odds of having low BMD increases with increasing serum levels of chemerin and decreasing serum levels of visfatin and calcium (**Table 3**).

**Table 3:** Logistic regression analysis showing factors associated with BMD.

Variables	B	OR	95% CI	*p-value*
**Visfatin (ng/mL)**	-0.009	0.991	0.982-1.000	0.040*
**Intelectin-1 (ng/mL)**	0.001	1.001	0.979-1.023	0.959
**Chemerin (ng/mL)**	1.017	2.765	1.025-7.462	0.045*
**Alkaline Phosphatase (U/L)**	0.007	1.007	0.997-1.017	0.154
**Calcium (mg/dL)**	-0.593	0.553	0.311-0.982	0.043*
**Phosphate (mg/dL)**	0.192	1.212	0.702-2.093	0.490

Dependent variable was BMD. Low BMD included both osteopenic and osteoporotic subjects. The independent variables were visfatin, intelectin-1, chemerin, alkaline phosphatase, calcium, phosphate. Model is adjusted for age and menopausal age. * p-value ≤ 0.050 is considered statistically significant.

The comparison of *RARRES2* rs7806429, *PBEF1* rs2302559 and *ITLN-1* rs2274907 genotypic and allelic frequencies among the study groups is presented in **Table 4**. For *RARRES2* rs7806429, PCR product sizes were 110 bp for C allele, 156 bp for T allele, and 210 bp for two outer primers. The χ^2^ test of rs7806429 variant in the studied subjects suggested that it did not deviated significantly from Hardy-Weinberg equilibrium (*p* > 0.050). No significant differences were observed in genotype frequencies between the groups concerning rs7806429 variant (*p* = 0.101). For *PBEF1* rs2302559, PCR product size was 247 bp. The χ^2^ test suggested that it did not deviated significantly from Hardy-Weinberg equilibrium (*p* > 0.050). No significant differences were observed in genotype frequencies between the groups concerning rs2302559 variant (*p* = 0.935). For *ITLN-1* rs2274907, PCR product sizes were 471 bp for A allele and 274 + 197 bp for T allele. The χ^2^ test suggested that it did not deviated significantly from Hardy-Weinberg equilibrium (*p* > 0.050). No significant differences were observed in genotype frequencies between the groups concerning rs2274907 variant (*p* = 0.365).

**Table 4:** Comparison of *RARRES2* rs7806429, *PBEF1* rs2302559 and *ITLN-1* rs2274907 genotypic and allelic frequencies among the study groups.

Genotypes		Normal	Osteopenia	Osteoporosis	*p*-value
		n (%)	n (%)	n (%)
***RARRES2* rs7806429**					
Genotype frequencies	TT	17 (23.6)	22 (31.0)	19 (19.0)	0.101
	TC	45 (62.5)	45 (63.4)	75 (75.0)
	CC	10 (13.9)	4 (5.6)	6 (6.0)
Allele frequencies	T	79 (0.55)	89 (0.63)	113 (0.57)	0.362
	C	65 (0.45)	53 (0.37)	87 (0.43)
***PBEF1* rs2302559**					
Genotype frequencies	TT	10 (13.9)	9 (12.7)	15 (15.2)	0.935
	TC	27 (37.5)	30 (42.3)	42 (42.4)
	CC	35 (48.6)	32 (45.1)	42 (42.4)
Allele frequencies	T	47 (0.33)	48 (0.34)	72 (0.36)	0.757
	C	97 (0.67)	94 (0.66)	126 (0.64)
***ITLN-1* rs2274907**					
Genotype frequencies	AA	31 (43.1)	20 (28.2)	37 (37.0)	0.365
	AT	39 (54.2)	49 (69.0)	58 (58.0)
	TT	2 (2.8)	2 (2.8)	5 (5.0)
Allele frequencies	A	101 (0.70)	89 (0.63)	132 (0.66)	0.408
	T	43 (0.30)	53 (0.37)	68 (0.34)

Chi square test was applied to compare frequencies.

The results of association analysis of *RARRES2* rs7806429, *PBEF1* rs2302559 and *ITLN-1* rs2274907 genotypes with their respective serum parameters and BMD at various sites in the studied subjects is shown in **Table 5**.

**Table 5:** Comparison of *RARRES2* rs7806429, *PBEF1* rs2302559 and *ITLN-1* rs2274907 genotypes with their respective serum parameters and BMD at various sites.

		RARRES2	Lumbar spine	Right femoral neck	Left femoral neck	Total hip
***RARRES2* rs7806429**	**TT** **n = 58**	0.16 (0.13 to 0.41)	-1.70 (-2.50 to -0.50)	-0.75 (-1.92 to -0.07)	-1.05 (-1.70 to -0.30)	-0.50 (-1.37 to 0.16)
	**TC** **n = 165**	0.16 (0.12 to 0.36)	-1.90 (-2.75 to -0.70)	-1.30 (-2.30 to -0.30)	-1.30 (-2.10 to -0.50)	-1.00 (-1.87 to 0.02)
	**CC** **n = 20**	0.15 (0.10 to 0.49)	-0.85 (-2.57 to 0.15)	-0.20 (-1.77 to 0.60)	-0.85 (-2.07 to 0.47)	-0.27 (-1.67 to 0.91)
	*p*-value*	0.708	0.143	0.033^a^	0.381	0.089
		**Visfatin**	**Lumbar spine**	**Right femoral neck**	**Left femoral neck**	**Total hip**
***PBEF1* rs2302559**	**TT** **n = 34**	6.47 (2.54 to 14.62)	-1.80 (-2.85 to -0.67)	-1.30 (-2.10 to -0.10)	-1.25 (-2.42 to -0.07)	-0.82 (-1.85 to -0.03)
	**TC** **n = 99**	6.85 (2.61 to 13.09)	-1.70 (-2.60 to -0.50)	-1.20 (-2.10 to -0.10)	-1.10 (-2.10 to -0.30)	-0.85 (-1.80 to 0.15)
	**CC** **n = 109**	4.86 (2.28 to 10.52)	-1.80 (-2.65 to -0.45)	-0.90 (-2.00 to 0.00)	-1.30 (-2.00 to -0.40)	-0.75 (-1.80 to 0.05)
	*p*-value*	0.176	0.782	0.413	0.734	0.931
		**ITLN-1**	**Lumbar spine**	**Right femoral neck**	**Left femoral neck**	**Total hip**
***ITLN-1* rs2274907**	**AA** **n = 88**	5.79 (1.50 to 13.11)	-1.60 (-2.70 to -0.42)	-1.05 (-2.27 to 0.02)	-1.35 (-2.1 to 0.00)	-0.90 (-1.98 to 0.03)
	**AT** **n = 146**	6.20 (2.34 to 17.75)	-1.80 (-2.60 to -0.70)	-1.00 (-2.10 to -0.20)	-1.10 (-2.00 to -0.40)	-0.77 (-1.51 to 0.15)
	**TT** **n = 9**	3.09 (2.24 to 10.78)	-1.60 (-2.95 to 0.40)	-1.60 (-2.00 to 0.00)	-1.50 (-2.00 to -0.60)	-1.60 (-2.30 to -0.27)
	*p*-value*	0.371	0.899	0.977	0.875	0.222

Comparisons are seen using independent sample Kruskal-Wallis test. Values are given in median (IQR). * p-value ≤ 0.050 is considered statistically significant. ^a^ significant between TC and CC only with adjusted significance of 0.05.

There was no significant difference in the serum levels of RARRES2 between rs7806429 genotypes (*p* = 0.708). There was significant difference in BMD at right femoral neck (*p* = 0.033) between rs7806429 genotypes. Multiple comparisons after dunn-bonferroni correction showed that BMD at right femoral neck was significantly low in the rs7806429 TC heterozygotes as compared to rs7806429 CC homozygotes.

Serum visfatin levels were low in rs2302559 CC homozygotes, but the difference was not significant (*p* = 0.179). No significant difference was found between BMD and genotypes of visfatin rs2302559 SNV.

Serum Intelectin-1 levels were low in rs2274907 TT homozygotes, but the difference was not significant (*p* = 0.371). No significant difference was found between BMD and genotypes of *ITLN-1* rs2274907 SNV (**Table 5**).

## Discussion

The study included postmenopausal non-osteoporotic, osteopenic and osteoporotic females. Osteoporosis increases the porosity of bones and increase risk of fragility fractures in postmenopausal females (19).

The adipokine RARRES2 can regulate both osteogenesis and adipogenesis. In the present study, serum RARRES2 levels were significantly higher in the osteoporotic group as compared to the non-osteoporotic and osteopenic postmenopausal females. In addition, serum RARRES2 levels were found to be independent negative predictor of BMD at the right and left femoral neck. Similar results were observed in other studies in which they found significantly increased levels of RARRES2 in osteoporotic patients (5, 20). In another study conducted on Chinese obese postmenopausal women, similar findings were observed. DXA scan was performed to assess the BMD of these subjects at lumbar spine and femoral neck. They exhibited a significant negative relationship of RARRES2 with BMD at lumbar spine. However, they could not established any relationship with femoral neck BMD. They further concluded RARRES2 as an independent negative predictor of BMD. The plausible cause of this negative correlation of chemerin with BMD was low-grade inflammation which affected bone mass in these individuals (21).

Chemerin is said to act as a negative regulator of osteogenesis. A study has shown an increase in expression of osteoblast marker gene and mineralization while decrease in adipocytes differentiation in RARRES2 and its receptor knock out mice (22). In addition, hematopoietic stem cells (HSC) also secretes RARRES2 and is involved in the process of osteoclastogenesis. The study showed that RARRES2 was neutralized by introducing a blocking antibody prior to osteoblast differentiation resulting in complete loss of the process of osteoclastogenesis. Reintroduction of RARRES2 by reversing the neutralization, initiated the process of osteoclast differentiation demonstrating that RARRES2 signaling is necessary to allow HSC separation into osteoclasts (23).

A recent study conducted on mice demonstrated that RARRES2 increases the bone resorption by increasing the activity of mature osteoclasts. Interestingly, to strengthen their findings they introduced RARRES2 antagonist along with its receptor antagonist (CCX832) and observed an inhibition in osteoclastic activity and process of bone resorption. Furthermore, they elaborated that the signaling pathway involved in the action of osteoclast through RARRES2 is regulated by the extracellular signal-regulated kinase 5 (ERK5). It is the phosphorylation of this protein that initiated the signaling cascade resulting in osteoclast activation (24).

In the present study, we also evaluated the possible association of *RARRES2* rs7806429 SNV with BMD in postmenopausal women. This is the first study to investigate such association. The results of association analysis of genotype rs7806429 polymorphism with serum RARRES2 levels and BMD at various sites showed that serum RARRES2 levels were not significantly associated with rs7806429 genotype distribution. While BMD at the right femoral neck was significantly high in the CC homozygotes. Further studies can clarify the role of rs7806429 SNV and RARRES2 in bone metabolism.

Visfatin, initially thought to be expressed only by the visceral adipose tissue is now known to be highly expressed by the bone marrow plasma, thus having a possibility of modulating bone metabolism (25).

In this study, serum levels of visfatin were lower in postmenopausal osteopenic and osteoporotic women as compared to non-osteoporotic women. Visfatin also appeared to be an independent positive predictor of BMD at lumbar spine and hip. The findings of this study are strongly supported by earlier studies, both *in vitro* and *in vivo*. Visfatin stimulates osteoblast proliferation, mineralization and increases the expression of type 1 collagen which is an important osteogenic marker (22). The inhibition of visfatin in mouse bone marrow mesenchymal stromal cells or visfatin knockout mouse showed reduced osteoblastogenesis, furthermore reducing serum levels of visfatin also decreases the activity of alkaline phosphatase and inhibits matrix mineralization resulting in reduce bone formation (11, 26). A study showed that serum visfatin levels have positive correlation with lumbar spine BMD and total BMD in survivors of acute lymphocytic leukemia (10).

There is quite scarce data available regarding the role of visfatin in osteoclastic function. However, most of the available data are supporting the findings of our study. Visfatin may negatively regulate the process of osteoclastogenesis. An *in vitro* study suggested the inhibitory role of visfatin on the differentiation of osteoclasts and generation of markers of osteoclastic activity, RANK, cathepsin-K and nuclear factor of activated T-cells c1 (NFATc1) (27). This finding was also observed in another *in vitro* study performed on bone marrow derived macrophages, where visfatin suppressed RANKL mediated osteoclastogenesis by suppressing the phosphorylation of several important signal transducers involved in the process of osteoclastogenesis. However, the activity of mature osteoclasts remained unaffected (28).

In contrast to our study, contradictory findings were observed in few other studies. Serum visfatin levels were negatively related with BMD (29). Serum visfatin levels were also measured in postmenopausal Iranian females and after performing multiple linear regression, they could not found any significant association of serum visfatin with bone markers (30).

Present study has also investigated the possible association of *PBEF1* genetic variant rs2302559 with BMD. This is the first study to investigate such association. The frequency of TT genotype of rs2302559 variant was less in postmenopausal osteoporotic females as compared to TC and CC genotype but the association was insignificant. We did not find any association of *PBEF1* genetic variant (rs2302559) with BMD and serum visfatin levels in postmenopausal females.

In this study, serum levels of Intelectin-1 were low in postmenopausal osteoporotic females as compared to non-osteoporotic females but Intelectin-1 was not a predictor of BMD at any site. Similar to our findings, a study found increase in levels of Intelectin-1 in postmenopausal women with osteoporosis and labelled it as a postmenopausal physiological compensation against bone loss (31). Intelectin-1 may have improved estrogen deficiency induced bone loss by inhibiting tumor necrosis factor-α (TNF- α) and further reduced the levels of leptin in obese Iranian women (32). Similarly, higher intelectin-1 levels were associated with lower BMD at lumbar spine only in non-diabetic postmenopausal females (33). Contradictory results were seen in older men with osteoporosis, in which it has negative correlation with BMD and bone turnover markers and this negative effect on bone was attributed to altered osteoblast differentiation *via* intelectin-1 (34).

*In vitro* studies have shown that Intelectin-1 is important in inhibiting the expression of pro-inflammatory cytokines IL-6, IL-8 and inflammatory mediators in the macrophages that are derived from the same cell lineage as osteoclasts. Intelectin-1 suppresses the accumulation of nuclear p65 and transfected nuclear factor (NF-κB) promotor activity thus reducing the activity of NF-κB pathway. They also directly reduce NF-κB activation. It also reduces the lipopolysaccharide (LPS) induced expression of the toll like receptor (TLR4) and myeloid differentiation primary response gene 88 (MyD88), thus inhibiting the TLR4/MyD88 signaling pathway (35). This pathway is important to induce the expression and secretion of various inflammatory cytokines that directly and indirectly enhance osteoclastogenesis. Intelectin-1 reduces oxidative stress by decreasing the levels of reactive oxygen species and increasing the antioxidant reduced glutathione (GSH). Intelectin-1 significantly increases the activity of nuclear translocation of nuclear factor erythroid 2-related factor 2 (Nrf2) thus increasing the transcription of cytoprotective proteins and inhibiting the expression of pro-inflammatory cytokines (35). All these effects are in favour of inhibiting osteoclastogenic effects and thus increased BMD.

The effects of Intelectin-1 on the osteoblasts showed the positive relation of Intelectin-1 with bone. It stimulates the proliferation of human osteoblasts by activating the phosphatidylinositol 3-kinase/serine-threonine protein kinase (PI3K/Akt) signaling pathway (36). Rao et al (2018), showed induction of inflammatory bone loss in Intelectin-1 knock out mice. They also demonstrated increase in pro-inflammatory cytokines, increase osteoclastogenesis, reduced osteogenic activity and enhanced bone destruction in the absence of Intelectin-1. Administration of recombinant intelectin-1 in the mouse model reduced the production of pro-inflammatory cytokines from macrophages thus reducing their pro-osteoclastic and anti-osteoblastic potential (37). Thus, all these findings strongly favour the bone-protecting role of Intelectin-1 against inflammation and osteoporotic bone loss.

Present study has also investigated the possible association of *ITLN-1* genetic variant rs2274907 with BMD. The frequency of TT genotype of rs2274907 variant was less in postmenopausal osteoporotic females as compared to AT and AA genotype but the association was insignificant. We did not find any association of *ITLN-1* rs2274907 genetic variant with BMD and serum Intelectin-1 levels in postmenopausal females. Further studies with bigger sample size are needed. Moreover, randomized controlled trials can be designed whose results can help in modifying the various factors associated with adipokines to reduce the risk of a highly prevalent disease, osteoporosis. The findings can also help in planning therapeutic and preventive aspects of a highly prevalent disease. We can go for genetic screening in offspring of the predisposed families, offer them early preventive counselling and measures resulting in higher quality of life of the sufferers. They can also act as a useful genetic marker for BMD and it would be possible to administer individual symptomatic treatment as well as early prophylactics of osteoporosis.

## Limitations

The limitations of the study include small sample size, a cross sectional design and inclusion of less parameters due to funding constraints. Bone turn over markers could have also been included.

## Conclusion

The adipokines RARRES2 and visfatin are associated with BMD. RARRES2 is independent negative and visfatin is positive predictor of BMD in postmenopausal females. BMD at right femoral neck was significantly low in RARRES2 rs7806429 TC heterozygotes.

## Data availability statement

The original contributions presented in the study are included in the article/supplementary materials. Further inquiries can be directed to the corresponding author.

## Ethics statement

The studies involving human participants were reviewed and approved by Ethics Committee of University of Health Sciences, Lahore. The patients/participants provided their written informed consent to participate in this study.

## Author contributions

SuT: Conception and design, acquisition, analysis, interpretation of data, drafted the manuscript. SaT: Acquisition, analysis of data, carried out the literature search, helped in drafting the manuscript. SK: Designed and supervised the research, analysis and interpretation of data, revised the manuscript critically for important intellectual content. SA: Data analysis, interpretation, drafted and revised the manuscript critically for important intellectual content. MB: Data analysis, interpretation, drafted and revised the manuscript critically for important intellectual content. All authors contributed to the article and approved the submitted version.

## Funding

The research is partially supported by grant from Higher Education Commission, Pakistan under grant number: 8530/Punjab/NRPU/R&D/HEC/2017.

## Acknowledgments

We are thankful to Prof. Dr. Khalid Parvez Lone (late) who supervised part of this research. We acknowledge the technical staff of Madina teaching hospital, The University of Faisalabad and Pakistan institute of nuclear medicine for helping in performing biochemical analysis and DXA measurements.

## Conflict of interest

The authors declare that the research was conducted in the absence of any commercial or financial relationships that could be construed as a potential conflict of interest.

## Publisher’s note

All claims expressed in this article are solely those of the authors and do not necessarily represent those of their affiliated organizations, or those of the publisher, the editors and the reviewers. Any product that may be evaluated in this article, or claim that may be made by its manufacturer, is not guaranteed or endorsed by the publisher.
